# Genetic algorithm optimization of broadband operation in a noise-like pulse fiber laser

**DOI:** 10.1038/s41598-023-28689-8

**Published:** 2023-02-01

**Authors:** Coraline Lapre, Fanchao Meng, Mathilde Hary, Christophe Finot, Goëry Genty, John M. Dudley

**Affiliations:** 1 Université de Franche-Comté, Institut FEMTO-ST, CNRS UMR 6174, 25000 Besançon, France; 2 State Key Laboratory of Integrated Optoelectronics, College of Electronic Science and Engineering, Jilin University, Changchun, 130012 China; 3 Photonics Laboratory, Tampere University, FI-33104 Tampere, Finland; 4 Université de Bourgogne, Laboratoire Interdisciplinaire Carnot de Bourgogne, CNRS UMR 6303, 21078 Dijon, France

**Keywords:** Nonlinear optics, Fibre lasers

## Abstract

The noise-like pulse regime of optical fiber lasers is highly complex, and associated with multiscale emission of random sub-picosecond pulses underneath a much longer envelope. With the addition of highly nonlinear fiber in the cavity, noise-like pulse lasers can also exhibit supercontinuum broadening and the generation of output spectra spanning 100’s of nm. Achieving these broadest bandwidths, however, requires careful optimization of the nonlinear polarization rotation based saturable absorber, which involves a very large potential parameter space. Here we study the spectral characteristics of a broadband noise-like pulse laser by scanning the laser operation over a random sample of 50,000 polarization settings, and we quantify that these broadest bandwidths are generated in only $\sim$ 0.5% of cases. We also show that a genetic algorithm can replace trial and error optimization to align the cavity for these broadband operating states.

## Introduction

Ultrashort pulse propagation in optical fiber is well known to lead to a wide range of complex nonlinear dynamics and processes such as modulation instability, soliton and self-similar evolution, and supercontinuum generation^1^. A comparably wide range of dynamics can be seen in modelocked fiber lasers, where the concept of the dissipative soliton in particular has provided a powerful framework within which to interpret the many different classes of laser operation that are observed^2–7^. From the point of view of applications, most studies of fiber lasers have focused on designs that produce highly regular pulse trains^8,9^, but there has also been extensive study from a fundamental perspective on laser instabilities. Indeed, the study of instabilities in fiber lasers have led to new insights into processes such as soliton molecule formation^10–12^, complex temporal pattern formation in lasers^13–15^, soliton rogue wave emergence^14,16^, as well as intermittence and aperiodic transitions between different regimes of stability and instability^17,18^.

A particularly unstable mode of fiber laser operation is the “noise-like pulse” (NLP) regime, where a large number (100–1000) of ultrafast pulses evolve randomly underneath a much broader envelope^19^. The NLP regime has been the focus of many recent experiments^20–27^, as well as demonstrations in applications such as low coherence metrology^28^ and material processing^29^. One recent study has reported an NLP laser around 1550 nm where the presence of highly-nonlinear fiber in the cavity leads to intracavity supercontinuum broadening with output spectra spanning 100’s of nm^30^. However, achieving these particular bandwidths was found to require highly precise trial and error optimization of the nonlinear polarization rotation based saturable absorber. In this paper, we report further experimental study of a broadband NLP laser, where we scan the laser operation over 50,000 polarization settings, and quantify that the broadest bandwidths are generated in only $\sim$ 0.5% of cases relative to the full sampled parameter space of laser operation. For systematic access to this broad bandwidth regime, we use a genetic algorithm that successfully replaces trial and error to automatically optimize the cavity alignment.

## Results

### Experimental setup and methods

**Figure 1:**
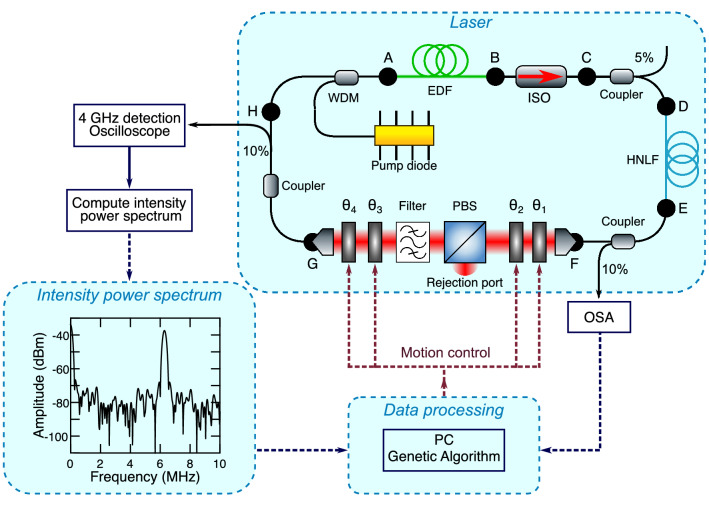
Schematic of the NLP laser and our experimental setup. Labels A-H refer to different fiber segments. The saturable absorber segment is between F and G. The figure also shows how feedback from the optical spectrum analyser (OSA) and the computed power spectrum from the oscilloscope (bottom left panel) are used as inputs to the genetic algorithm for laser optimization. *ISO* isolator; $\theta _1$ and $\theta _4$ quarter-wave plate, $\theta _2$ and $\theta _3$ half-wave plate; *PBS* polarizing beamsplitter, *EDF* Erbium-doped fiber, *HNLF* highly nonlinear fiber, *WDM* wavelength-division multiplexer.

Figure 1 shows the experimental setup, which is based on the system previously described in Ref.^30^. The laser is a unidirectional ring cavity and uses non-polarization-preserving fiber. Fiber lengths and parameters are as follows. Segment AB consists of 11 m of Erbium-doped fiber (EDF), segment BD consists of 1.8 m of standard single mode fiber (SMF-28), segment DE consists of 12 m of highly nonlinear fiber (HNLF), and segments EF and GA consist of 4.3 m and 3.1 m of SMF-28 respectively. The EDF has dispersion parameter $\beta _2 = +40 \times 10^{-3}\,{\text{ps}}^2\,{\text{m}}^{-1}$, and nonlinear parameter $\gamma = 6.0 \times 10^{-3}\,{\text{W}}^{-1}\,{\text{m}}^{-1}$. The SMF-28 dispersion parameters are $\beta _2 = -21.7\times 10^{-3}\,{\text{ps}}^2\,{\text{m}}^{-1}$, $\beta _3 = +86.0 \times 10^{-6}\,{\text{ps}}^3\,{\text{m}}^{-1}$, and nonlinear parameter $\gamma = 1.1 \times 10^{-3}\,{\text{W}}^{-1}\,{\text{m}}^{-1}$. The HNLF has dispersion parameters $\beta _2 = -5.23\times 10^{-3}\,{\text{ps}}^2\,{\text{m}}^{-1}$, $\beta _3 = +42.8\times 10^{-6}\,\text{ps}^3\,\text{m}^{-1}$ and nonlinear parameter $\gamma = 18.4 \times 10^{-3}\,\text{W}^{-1}\,\text{m}^{-1}$. All dispersion and nonlinearity parameters are specified at 1550 nm, and the net cavity dispersion is + 0.17 ps$^2$.

A $\sim$ 40 cm bulk-optics free space segment FG includes a nonlinear polarization rotation based saturable absorber. As shown in the figure, the input to the saturable absorber encounters sequentially a quarter-wave plate (angle $\theta _1$), a half-wave plate (angle $\theta _2$), a polarizing beamsplitter (PBS), a supergaussian filter, a half-wave plate (angle $\theta _3$), and a quarter-wave plate (angle $\theta _4$). This configuration allows a polarization-dependent loss to discriminate between pulsed and continuous wave (CW) operation in a fiber laser^31^. The waveplates were placed in motorized rotation stages (Thorlabs PRM128) controlled by programmable DC servo motors (Thorlabs KDC101). The spectral filter after the PBS (Andover (155FSX10-25) had 10 nm bandwidth (FWHM) and 80% peak transmission, and is used to control the bandwidth of the pulses reinjected into the EDF^8,32^. The EDF was co-directionally pumped at 976 nm, and noise-like pulsed behaviour was observed at all values of pump power above the 40 mW pump threshold where pulsed laser operation is first observed. The laser output was sampled at various points as shown in the figure for spectral and temporal characterisation as described below. The cavity repetition rate is 6.28 MHz (roundtrip time of $\sim$ 159 ns.)

## Statistical analysis of NLP bandwidth

At all pump powers, the spectral characteristics were found to be sensitively dependent on the precise settings of the waveplates in the saturable absorber, which is of course a well-known property of lasers modelocked through nonlinear polarization rotation^33^. To investigate this further, we set the pump power at 195 mW (well above the threshold of $\sim$ 40 mW) and then we varied the angular orientations of the waveplates to effectively characterize the different states of laser operation over their full ranges. This was performed used Latin hypercube sampling to generate 50,000 quadruples ($\theta _1,\theta _2,\theta _3,\theta _4$) to randomly sample the angular settings of the quarter-wave plates ($\theta _1,\theta _4$) over the range $[0,\pi ]$, and the half-wave plates ($\theta _2,\theta _4$) over the range $[0,\pi /2]$. For each setting of waveplate angles, we measured the average spectrum of the laser output after the HNLF using an integrating OSA (Anritsu MS9710B) over a span of 1500–1750 nm. We note that the spectral bandwidth of the laser can actually extend out to above 2100 nm at the -30 dB level because of supercontinuum broadening in the HNLF^30^, but measuring this full wavelength span actually requires concatenation of spectra from two detectors. However, as we will see below, we found that simply optimizing the spectral width as measured up to 1750 nm using the Anritsu MS9710B was sufficient to identify the targeted broadband operating regime. (Note that once optimized, we characterized the full operating bandwidth using an Ocean Optics NIRQuest512 spectrometer that allowed measurements over an extended spectral range.) We also used a fast photodiode (Thorlabs DET08CFC/M-5 GHz) and 4 GHz oscilloscope (Rohde & Schwarz RTO2044) from which an FFT based intensity power spectrum was computed (see Fig. 1). As we discuss below, we can extract a contrast metric from fundamental harmonic peak in this spectrum to provides a convenient measure of operation in the NLP regime.

**Figure 2:**
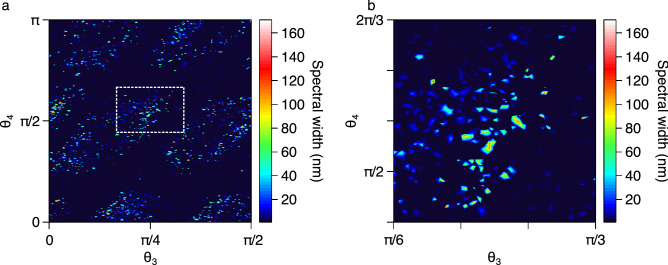
(**a**) False-color plot mapping how the − 20 dB spectral bandwidth of the laser output varies as a function of waveplate angles $\theta _3$ and $\theta _4$ with pump power at 195 mW. (**b**) An expanded view over the region indicated by the white square in (**a**) to illustrate the rarity of operating states of bandwidths exceeding $\sim$ 100 nm (corresponding to red regions in the plot).

To illustrate the different regimes of laser behaviour that are observed as a function of the multi-dimensional space of waveplate orientations, we plot in Fig. 2a the measured spectral bandwidth (at the − 20 dB level) as a function of waveplate angle pairs $\theta _3,\theta _4$ over their full ranges. Note that the bandwidth-dependence on waveplate position is in a four-dimensional space such that the results in the figure correspond to a projection through the $\theta _3,\theta _4$ plane in the space where all four angles are being simultaneously scanned. In other words, the angles $\theta _1,\theta _2$ are themselves being varied at each point plotted in the $\theta _3,\theta _4$ plane. Note that similar results are obtained when plotting against other polarization angle pairs.

The results in Fig. 2a reveal the essential features of the dynamics, and show that the noise-like pulse states of bandwidth exceeding $\sim$ 20 nm exists in relatively well-defined “islands” within a broader state space where the laser operation is associated with narrowband or quasi-continuous wave operation. The broadest bandwidths exceeding $\sim$ 100 nm occupy only a very small fraction of observed output states and are observed for very particular combinations of waveplate positions as shown in the expanded view in Fig. 2b. The average laser output power (measured after point C in the cavity) was typically $\sim$ 3 mW for operating points within the broadband islands and typically $\sim$ 3.5 mW in the narrowband regimes. Note that the randomness of these locations in the polarization space is attributed to the random birefringence in the cavity due to the use of non polarization-preserving fiber.

**Figure 3:**
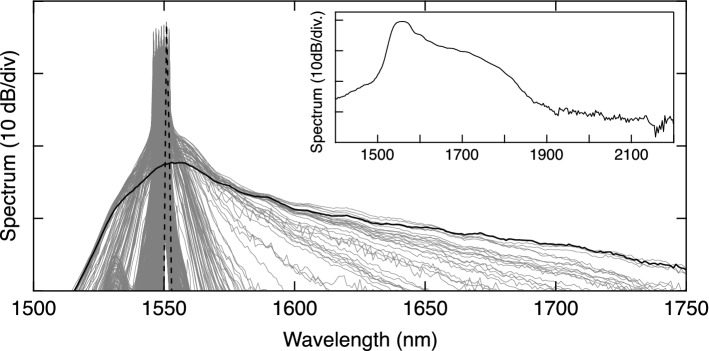
2000 spectra measured with the Anritsu OSA, selected randomly from the full scan (gray curves). Specific examples of a narrowband spectrum (dashed black line) and a broadband spectrum (solid black line) are also shown. The inset plots a broadband spectrum measured using the NIRQuest spectrometer to show spectral extension above 1750 nm.

The variation in spectral properties across the polarization space is also shown in Fig. 3. Here the gray curves superimpose 2000 spectra measured with the Anritsu OSA, selected randomly from the full parameter scan. The figure highlights one narrowband spectrum associated with quasi-continuous wave operation (dashed black line) as well as a broadband spectrum observed in the NLP regime (solid black line). The inset plots a typical broadband spectrum measured using the NIRQuest spectrometer to show the spectral extension above 1750 nm.

**Figure 4:**
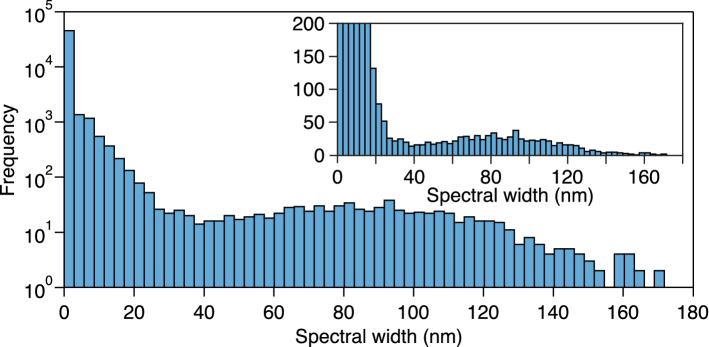
Histogram of the measured − 20 dB bandwidths from the full cavity scan of 50,000 polarization settings of the saturable absorber. The main plot uses a logarithmic scale for the vertical axis whereas the inset shows an exploded view on a linear scale to illustrate the long-tailed nature of the distribution.

To discuss the statistical properties of the different regimes of laser operation seen in this scan, Fig. 4 plots a histogram of the − 20 dB laser bandwidth computed from the full set of 50,000 spectral measurements. The main plot uses a logarithmic vertical axis, whereas the inset shows an exploded view on a linear scale. The distribution is strongly long-tailed and indeed, only around 1000 measured spectra (2% of the total measured dataset) have a bandwidth exceeding 20 nm. Indeed, operation at a bandwidth greater than 20 nm can be taken as a useful indicator of entry into the NLP regime, as this is where points begin to cluster into the localized regions shown in Fig. 2. The broadest bandwidths constitute an even smaller fraction of the operating states, with only $\sim$ 0.5% of the measured bandwidths exceeding 100 nm at the − 20 dB level.

## Genetic algorithm

Locating the optimal polarization orientation of a NLP laser is usually performed by trial and error, but we now discuss how this process can be replaced by a genetic algorithm (GA). Our use of GA optimization rather than other gradient methods here is motivated for several reasons. Firstly, we note that the comparison between GA and other gradient methods remains an active topic of research in machine learning, and both techniques are known to have advantages and disadvantages^34^. This said, gradient-based methods are known to show particular difficulties with noisy objective function spaces and inaccurate gradient calculations, and for the particular regime that we seek to optimize (from chaotic non-modelocked to broadband modelocked operation) a GA method is more suitable. We also remark that GA techniques have been previously applied to other passively modelocked fiber lasers^35,36^, and a further specific motivation of our work was to test the performance of a GA for a noise-like pulse laser. By keeping the optimization method the same as previous studies, it was possible to confirm the specific utility of the GA approach for a laser with complex broadband intracavity dynamics.

Our GA is summarised as follows. Each particular set of 4 waveplate orientations ($\theta _1,\theta _2,\theta _3,\theta _4$) describes an “individual,” with the setting of each waveplate constituting a “gene.” We consider a population of 50 individuals with initial genes (angles) that are randomly selected and, based on the laser output characteristics, we calculate (and minimize) an objective function defined in order to discriminate between broadband NLP operation and other modes of narrowband CW or quasi-CW emission in the cavity. In this context, we note that it is essential we scan over all four waveplates. For example, if we only varied $\theta _3$ and $\theta _4$ without also varying $\theta _1$ and $\theta _2$, there is no guarantee that we would reach the broadest bandwidth regime as this would depend critically on the particular fixed $\theta _1$ and $\theta _2$ settings. It is also necessary to scan the waveplates over their full angular ranges. Specifically, although the localized islands seen in Fig. 2a might suggest that we could locate the optimal bandwidth by scanning a limited angular range, the particular polarization settings associated with broadband operation are highly sensitive to variations in random fiber birefringence due to environmental factors. As a result, a reduced angular search space would be valid only for a finite period of time. For the most general utility of the technique, searching over the full parameter space is required.

Our GA aims to minimize the compound objective function defined as: $C = S_{\text{peak}} /S_{\text{ref}} - \delta \lambda / \delta \lambda _{\text{ref}}$ where the first term selects for the appearance of a strong fundamental harmonic peak in the intensity power spectrum (see Fig. 1), and the second term selects for the generation of a broadband optical spectrum. In particular concerning the first term, $S_{\text{peak}}$ is the mean value of the fundamental harmonic peak computed within $\pm 200$ kHz of the laser repetition frequency, and the reference value of $S_{\text{ref}} = -\,35$ dBm. When the laser is poorly modelocked, the value of $S_{\text{peak}}$ approaches the spectral noise floor (around $-80$ dBm) such that the contribution of the first term to the objective function is large. In contrast, the presence of a strong harmonic peak associated with modelocking results in $S_{\text{peak}}$ increasing towards the reference value so the contribution of the first term to the objective function decreases. Concerning the second term, $\delta \lambda$ is the root mean square bandwidth of the spectrum measured on the OSA, and $\delta \lambda _{\text{ref}}=10$ nm is a reference value. Since the second term is associated with a minus sign, increasing the bandwidth relative to the reference acts to minimise the objective function. Since the objective function is based on two components (the harmonic peak and the bandwidth), it is important that these are appropriately weighted. Such weighting is included through the reference parameters $S_{\text{ref}}$ and $\delta \lambda _{\text{ref}}$, and the values given above were based on extensive testing to ensure that the GA did not display divergent behaviour. Indeed, we tested the GA over more than 100 generations and did not find any evidence of divergence. We also note that any potential bandwidth-related divergence will be limited by the transmission window of the various elements in the system (the SMF28 and HNLF fiber, couplers etc.) which will result in a finite minimum value of the objective function.

The algorithm iterates using standard GA techniques^37,38^. For each subsequent generation, 3 individuals are selected as elite and propagate deterministically through to the next generation without change of genes. A crossover fraction of 0.7 is then applied to the non-elite individuals to generate 33 individuals in the following generation that are constructed combining genes from individuals with the lowest fitness values. The remaining individuals are constructed from mutation. We then iterate and monitor the evolution of the fitness values over a number of generations. All coding was performed in MATLAB using the Global Optimization toolbox. The elite and crossover fractions were based on typical algorithm parameterisations, but the GA was found to work over a wide range of values.

**Figure 5:**
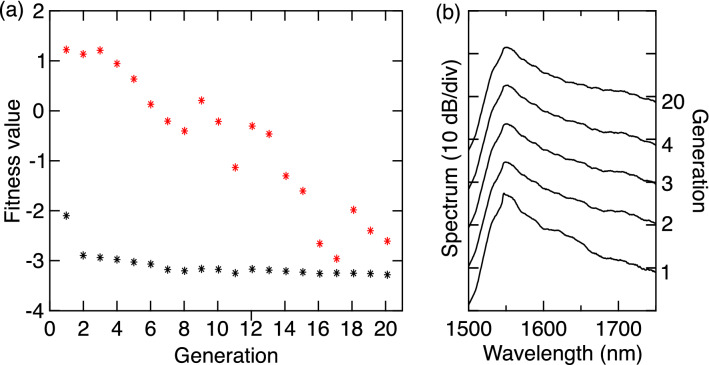
(**a**) Evolution of the objective function over 20 generations of the genetic algorithm. At each generation, we show the mean of the population (red stars) and the particular individuals corresponding to the minimum fitness values (black stars). (**b**) For the data in (**a**) we show the spectrum of the best individual for the generations as indicated on the right hand axis.

Typical results of the GA optimization procedure are shown in Fig. 5. Specifically, over 20 generations, Fig. 5a plots the mean objective function computed over the 50 individuals in the population (red stars), as well as the minimum objective function for the best individuals (black stars). For completeness, Fig. 5b shows the spectra of the best individuals for selected generations as indicated on the right hand axis. The GA evolution shown in the figure reveals that we rapidly enter into a regime of convergence. Indeed, for these particular results, the algorithm appears to identify the optimal regime after only 2 generations, although the precise evolution for any particular experiment does depend on the initial genes that are selected randomly. Nonetheless, additional testing revealed that between 2 and 4 generations to enter into the optimal regime was typical.

We also remark that there is some small variation in the best value of the objective function which is attributed to residual hysteresis in the laser operation, a well known effect in such lasers^39^. Once the GA has converged, the spectral characteristics are visually identical to the solid black curve shown in Fig. 3, associated with − 20 dB bandwidths of $\sim$ 170 nm. Note that we performed extensive tests with multiple runs of the GA starting from different initial conditions, and they all yielded similar results to those in Fig. 5 with comparable − 20 dB bandwidths after optimization. We also tested the algorithm performance for populations in the range 20–100, and the population of 50 for which we show results here was found to reliably yield convergence to the broadband regime for any initial condition. No significant improvement in the results was obtained using more than 20 generations.

## Discussion and conclusions

As a general conclusion, these results provide further insight into the operation of NLP lasers, and provide an additional example of the utility of machine learning techniques in optimizing laser operation in complex regimes. Our experiments have clearly shown the strong dependence of NLP operation on the precise settings of the saturable absorber polarization settings, and directly revealed the very small fraction of the full parameter space that is associated with the broadest bandwidths. However, despite this complexity, automatic optimization using a genetic algorithm has been shown to effectively locate the broadest bandwidth operating regimes associated with − 20 dB bandwidths of $\sim$ 170 nm within only a few generations (only a few minutes of alignment.)

These results represent a significant improvement compared to trial and error (human) optimization where the need to monitor multiple instruments whilst adjusting four waveplates results in typical alignment times of several hours. In this context, we anticipate that the search time for the genetic algorithm could be further reduced with improved instrumentation. Specifically, our setup suffered from two particular bottlenecks: (i) the use of a scanning OSA to measure the spectrum for each individual in the population and at each generation; (ii) the use of bulk motor-controlled rotation stages to modify the waveplate orientations in the saturable absorber for each individual in the population and at each generation. Whilst this equipment choice was convenient for experimental flexibility in a laboratory setup, there are alternatives available that would result in significantly faster acquisition and adjustment. For example, it could be possible to use a long wavelength filter and a simple photodetector to optimize the extension of the output spectrum, and an integrated polarization controller could replace the bulk system that we employed. In an industrial setting, optimization times of less than a minute should be achievable.

## Data Availability

Data are available from the corresponding author upon reasonable request. All code used in the manuscript was the standard GA suite within the MATLAB Global Optimization toolbox.
